# Interexaminer reproducibility for subjective refractions for an ametropic participant

**DOI:** 10.1136/bmjophth-2021-000954

**Published:** 2022-05-11

**Authors:** Solani David Mathebula, Alan Rubin

**Affiliations:** 1 Optometry, University of Limpopo, Sovenga, Limpopo, South Africa; 2 Optometry, University of Johannesburg, Johannesburg, Gauteng, South Africa

**Keywords:** Optics and Refraction

## Abstract

**Objective:**

To investigate interexaminer reproducibility of non-cycloplegic subjective refractions. Subjective refractions are frequently determined, and it is important to know whether differences in refractive state over time constitute meaningful, non-random change.

**Methods and analysis:**

Fifty registered and experienced (≥5 years) optometrists from a single geographic region performed non-cycloplegic subjective refractions for a participant with moderate left eye(OS) to severe right eye (OD) ametropia. Subjective refractions were transformed to power matrices for analysis with stereopairs, distribution ellipsoids and polar profiles of variance of dioptric power. Absolute 95% limits of reproducibility ( $1.96\left(\sqrt{2}\right)$ (SD)) for excesses of subjective refractions for the right and left eyes separately from mean subjective refractions were determined.

**Results:**

Mean subjective refractions were −7.68–4.50×10 and −4.59–1.85×178 for the right and left eyes, respectively. The 95% absolute reproducibility limits for the stigmatic coefficients (spherical equivalents) were ≤1.71 D and ≤0.75 D for the right and left eyes, but corresponding limits for astigmatic coefficients were smaller (≤0.69 D).

**Conclusion:**

Removal of possible outliers for OD and OS, respectively, reduces the absolute 95% reproducibility limits for the stigmatic and astigmatic coefficients to ≤0.97 D and ≤0.49 D, thus improving interexaminer reproducibility. However, these results suggest caution with analysis of refractive data where subjective rather than objective methods are applied for longitudinal and epidemiological studies.

**What is already known on this topic?**

- The core function of optometrists is the prescription of refractive compensation. However, there is a lack of evidence-based research on reproducibility of subjective refraction and criteria for prescribing a refractive compensation. Subjective refraction is the benchmark against which all refractive methods are measured.

**What this study adds?**

- Spherocylindrical refractive data were transformed to power vectors and matrices, which has three dioptric components. The findings of this paper suggest that refractions performed by multiple optometrists on a single ametropic patient may differ in the stigmatic (sphere-equivalent) by 1.2 D in a moderate to severe compound myopic astigmatism and by 0.5 D in a mild compound myopic astigmatic eye. The 95% reproducibility limits for stigmatic data reduced to ≤0.6 D after removal of possible outliers. The reproducibility is the value within which the absolute difference between test results obtained under reproducibility conditions may be expected to lie with a probability of 95%.

**How this study might affect research, practice or policy?**

- In a legal dispute, the question may arise as to the circumstances under which the ocular refraction measured for a given patient can be regarded as being wrong or incorrect when the examiner faces possible liability in the event of a complaint.
- The reproducibility of subjective refraction or relating to other methods has profound implications for the effective analysis of refractive data obtained by multiple optometrists over the course of longitudinal or epidemiological studies.

## Introduction

Despite substantial advancements in methods for objective refraction, determination of the subjective (manifest) refraction remains the gold standard for measurement of refractive error. Subjective refraction (SR), which is the determination of the refractive error of the two eyes with involvement by the patient, is an important part of clinical eye care to determine compensatory lenses for optimum vision. Subjective refraction is also important because patients largely judge their quality of care received on the clarity and comfort of their compensatory lens prescriptions. Importantly, SR varies over time and in relation to many factors.^1^ Similar to visual acuity, refractive error also varies with changes in ocular accommodation and pupil diameter. Cooperation and communication between patients and examiners^1^ and ocular and general health can also affect SR.

Goss and Grosvenor^2^ in a review of reliability of refraction reported that intraexaminer and interexaminer reliability of SR was near 80% agreement within ±0.25 D and near 95% agreement within ±0.50 D for sphere and cylinder power. There is an assumption that SR provides an estimate of refractive state that is at once accurate and precise.^3–6^ Precision (*repeatability* and *reproducibility*) of refractive error measurement is important for both clinical decisions as well as research applications. It is also necessary to understand whether small differences from one examiner to another constitutes real change in refractive error. In the context of refractive errors, *repeatability* refers to several or repeated refractive measurements of SR by one examiner on the same participant when all other factors are assumed constant (meaning SR is measured under the same conditions).^3^ *Reproducibility* refers to the variability of SR by several examiners over time and the agreement between their findings is assessed.^4^ Variability in SR relates to many factors such as the examiner/s, instrument calibration, environment or time.^1^ Reproducibility establishes whether two or more examiners using the same (or similar) method of measurement can obtain the same or similar results. Repeatability and reproducibility are measures of precision or closeness of agreement and whether over time a given method of measurement accurately measures what it aims to measure.^3^

While studies^7–10^ mostly have examined the repeatability of objective refraction (such as retinoscopy, autorefraction or refractions from wavefront technology), data concerning the reproducibility of SR under masked conditions, where the examiners are unaware of the refractive results as measured by others, are limited. However, MacKenzie^11^ investigated the reproducibility of the spherocylinder prescriptions for an asymptomatic 29-year-old patient with near-emmetropia using 40 experienced optometrists as the sample. The study found that refractions performed by multiple optometrists (N=40) on a single eye will differ in the spherical equivalent by ≈ 0.78 D on average with a 95% limit of agreement of −1.38 and −0.28 D. Rosenfield and Chiu^10^ reported the 95% limit of agreement for SR to be ±0.29 D, which suggests that SR is accurate to about ±0.25 D and that a change of more than 0.50 D or more should be viewed as clinically significant. Zadnik *et al* 7 carried out repeatability studies on repeated (two) subjective refractions per eye with 40 participants and reported that 95% limits of agreement were 0.63 D from the mean SR.

Several studies^6–10^ have described the variation of refractive error measurement separately in terms of spherical (*F*_s_ or *S*) and cylindrical (*F*_c_ or *C*) powers, which resulted in inaccuracies because of the three-dimensional or multivariate nature of refractive power; one cannot simply separate *F*_s_, *F*_c_ and the axes (*A*) of the cylinders concerned. Suitable methods for the complete analysis of dioptric power and SR have been developed and are used widely by clinicians and researchers.^12–28^ MacKenzie^11^ used power vectors^23 26^ and matrices^14 15 19^ (**t** and **F** respectively) to evaluate the reproducibility of SR (for one eye of a healthy young male participant) as determined by 40 experienced optometrists in the UK. The eye was near-emmetropic and as is standard clinical practice in the UK, mostly the optometrists used trial frames and loose trial lenses for SR, whereas in the present study, mainly phoropters were used with trial frames and lenses applied where optometrists felt that was required (eg, for the right eye here with greater ametropia).

Sometimes in legal disputes, such as relating to motor vehicle accidents, a question may arise as to the circumstances under which the ophthalmic refraction measured for a given patient can be regarded as being incorrect and thus issues such as validity, reliability and reproducibility become relevant. There have also been discussions among researchers into the reproducibility of ophthalmic refractive results and ‘What might happen if a patient has been consulting the same examiner for years and decides to consult another qualified and possibly equally skilled examiner? Will the new refraction result be the same or different from previous results?’ Presently, there are no ophthalmic refractive methods to replace the gold-standard SR, although some have argued the case for measurements based on automated methods such as from autorefraction or wavefront aberrometry.^8 29–31^

Mostly studies that reported on the variability of refractive error measurements were designed to evaluate the performance and repeatability of autorefractors.^6–10 25^ Although such studies provided valuable information on the repeatability of SR, they were typically based on very few measurements of SR per participant and usually only for one eye per participant. Moreover, studies investigating the reproducibility of refractive error measurements are relatively scarce. Possible limitations of these studies were that results were from only two or three examiners and the examiners usually were unmasked as to the results of previous refractions. The analyses for some studies were possibly incomplete or erroneous as often only spherical equivalents were analysed and cylinder powers and axes were sometimes ignored.^6–10^

Consequently, there is a lack of evidence-based research on reproducibility of refractive error measurement and particularly relating to SR. The purpose of this study was to evaluate the reproducibility of spherocylindrical SR of a 25–30-year-old participant using measurements collected from 50 independent and fully qualified optometrists with clinical experience of ≥5 years. The optometrists were unaware of previous SR for the participant or of SR as determined by other examiners in the study.

## Materials and methods

A symptomatic participant was examined by 50 experienced optometrists selected purposefully from clinical practices in and around Polokwane in the Capricorn District of Limpopo Province, South Africa. The optometrists were masked as to the nature of the study and were unaware other optometrists had examined the same participant. To avoid possible biases, such as spending extra time when refracting the specific participant, the optometrists were unaware that their SR would be used for the study. The participant was in an excellent health and free of systemic or ocular diseases, except for being myopic, astigmatic and anisometropic (the right eye had greater compound myopic astigmatism (CMA) and this was considered, *a priori*, to be an interesting issue in terms of potential differences in ease of measurement of the subjective refractions to be obtained from the different optometrists involved in the study).

The participant independently underwent a comprehensive eye examination by 50 optometrists that did all procedures deemed necessary to address the chief complaint of the participant, which was simply *blurred distance vision* after recently losing her previous spectacles. The unaided distance visual acuities for right and left eyes were 6/150 and 6/60, respectively. With pinholes, these unaided distance VA improved to 6/9 and 6/6 in the right and left eyes. The slight reduction of monocular visual acuities with pinholes was attributed to anisometropic amblyopia and microtropia was not present.

Unlike the study by MacKenzie^11^ where trial frames and lenses only were used for SR, all optometrists in this study used their phoropters to determine the participant’s refractive state (SR). However, some might have also confirmed their phoropter SR with trial frames and loose trial lenses, as this is what most optometrists in the region concerned probably would do with patients with moderate or severe CMA and especially with relatively large cylinder magnitudes as for the one eye of this participant. Scripts for SR were independently given to the participant by each optometrist concerned and these measurements (SR) were used for further analysis. All SR were obtained without cycloplegia.

### Statistical analysis

Three numbers represent spherocylindrical powers: the sphere (*F*_s_ or *S*) and cylinder (*F*_c_ or *C*) powers and the orientation (axis, *A*) of the cylinder. Although this representation of dioptric power for refractive state is satisfactory for clinical purposes, it is mathematically unorthodox and other scientists, mathematicians or statisticians would not know how to work with these quantities. This necessitates a transformation of the data using the previously mentioned power vectors^23 26^ or matrices.^12 13^ Thereafter, the refractive data here were analysed using specially developed software based on Matlab, V.20 (The MathWorks, Natick, Massachusetts) or earlier versions.

Briefly, each spherocylindrical SR was transformed^13^ from clinical notation (*F*_s_, *F*_c_ and *A* where the reference meridian for axis, *A*, is horizontal) to a power matrix (**F** *_i_* where *i*=1:50) or, where applicable, to symmetric power vectors in **f**-notation (in some plots to follow **h**-notation is instead used—see reference 13 and later for the necessary equations for this coordinate vector):

(1) $$F_{i}=\left(\begin{array}{cc} f_{11} & f_{12}\\ f_{21} & f_{22} \end{array}\right)D.$$

Since **F** is symmetric, *f*_12_ = *f*_21_ and only three entries are unique. Coefficients from **F** are also determined for some graphical and quantitative applications. These coefficients are:

(2) $$F_{I}=0.5(f_{11}+f_{22})$$

(3) $$F_{J}=0.5\left(f_{11}-f_{22}\right)$$

and

(4) $$F_{k}=0.5(f_{21}+f_{12}).$$

Vector **f** is indicated below and relates also to Thibos *et al* 26; here we use vector **f** (that closely relates to **t** as per Thibos *et al*):

(5) $$f=\left(\begin{array}{c} F_{I}\\ F_{J}\\ F_{K} \end{array}\right)=\left(\begin{array}{c} M\\ J_{0}\\ J_{45} \end{array}\right)=t$$

where *F*_I_ (=*M*) is the scalar (or spherical) power and *F*_J_ (=*J*_0_) and *F*_K_ (=*J*_45_) are, respectively, the orthoantistigmatic and oblique antistigmatic powers. (The term antistigmatic (or antiscalar) is synonymous with Jackson cross cylinder (JCC) and each SR is, thus, represented scientifically with a scalar and two antistigmatic powers. The terms Jacksonian (or antistigmatic) are also sometimes used here for JCC. (Jacksonian powers can be represented conventionally, with power vectors or with 2×2 power matrices.)

With these vector or matrix forms, the mathematics and statistics of multivariate quantities were employed and, for example, matrices were used to plot points for SR in a 3-space called symmetric dioptric power space (SDPS). All spherocylindrical refractive measurements for both eyes of the participant were transformed and represented in SDPS. Thus, representation in SDPS of the 50 measures for SR for each eye becomes possible in terms of three independent or orthogonal powers, namely, a scalar power and two Jacksonian (antistigmatic) powers. The scalar power (*F*_I_) is identical to the spherical equivalent (*F*_ns_). The two Jacksonian powers include one with its power meridians along 0° and 90°, and the other with its power meridians along 45° and 135° and, as mentioned, are known as the orthoantistigmatic and oblique antistigmatic powers, respectively. These two antistigmatic powers (*F*_J_ and *F*_K_) are identical to *J*_0_ and *J*_45_^,^ respectively.

In this study, Mahalanobis distances (MD) were used for the identification of outliers, within the samples of 50 subjective refractions per eye. MD is an effective method that finds statistical distance in terms of SD between any two points in multivariate data to detect outliers.^31–33^ It is unitless in terms of SDPS (see Equation 6 where the units, $\left(\frac{D^{2}}{D^{2}}\right),$ cancel out). Meridional plots (see the online supplemental file to the paper) were also used to understand sample normality and, for example, of sample skewness and kurtosis. In this study, reproducibility was interpreted as the differences for SR as obtained by the 50 different optometrists over the time involved for the study and for the SR to be measured. Excesses (differences of each SR as subtracted from the mean SR ( $\overset{-}{SR}$ ) for the sample of 50 optometrists) for both OD and OS were used to calculate 95% limits of agreement (±1.96(SD)) (these are what we might obtain via Bland-Altman plots of means vs differences) and 95% absolute reproducibility limits (1.96( $\sqrt{2}$ )(SD)) for the scalar and antistigmatic (or antiscalar) coefficients of power, *F*_I_, *F*_J_ and *F*_K._ The 95% absolute reproducibility limit is the maximum expected difference in SR that might be obtained by the different examiners concerned, whether 2 or 50 as here or 40 as for MacKenzie.^11^

10.1136/bmjophth-2021-000954.supp1 Supplementary data

In terms of column coordinate vector **f,** the sample mean $\overset{-}{f}$ (see Equation 5) and the sample variance-covariance S the Mahalonobis distances for *i*- measures is:

(6) $$MD_{i}=\sqrt{{\left(f_{i}-\overset{-}{f}\right)}^{T}S^{-1}\left(f_{i}-\overset{-}{f}\right)}.$$

where the sample variance-covariance matrix is^13^:

(7) $$S=\sum_{i=1}^{N}\left(f_{i}-\overset{-}{f}\right)\left({\left(f_{i}-\overset{-}{f}\right)}^{T}/\left(N-1\right)\right)$$

(In Eq. 7, the sample size is *N* and the units are squared dioptres. Stigmatic or antistigmatic SD or SD with units of dioptres can be obtained via the square roots of the respective entries along the diagonal of S —see Equation 6 and Equation 7 above). By contrast, an Euclidean distance between two powers (say, **f**_p_ and **f**_q_) in SDPS is^13^:

(8) $$||f_{q}-f_{p}||=\sqrt{{\left(f_{q}-f_{p}\right)}^{T}\left(f_{q}-f_{p}\right)}$$

and the units would be dioptres. So, MD and Euclidean distances (or lengths) or residuals are not quite the same as is seen by comparing Equations 6 and 8.

In statistics, a *residual* is the difference between an observation (such as SR here) and an average or mean value for a sample. Residuals are commonly applied in statistical modelling involving regression and Analysis of variance (ANOVA) where they are important in terms of understanding sample variation.^34^ Sometimes, the term *error* is used in statistics instead of residual to compare the observed and true values of a variable, although the true value may sometimes be unknown and hence the use of an estimator of the true value such as a sample mean for comparative purposes. The term residual can be applied to a simple difference (or excess) in SR in relation to the mean SR for a sample or it could refer to a dioptric difference in SDPS (such as the Euclidean length of a comet or part of a starburst), where it would be the same as an Euclidean difference (see Eq. 8). To avoid confusion here, we will reserve its use for the latter and use the term excess or difference below for a comparison of the individual SR to the sample means.

So, in SDPS, a residual is similar to an MD in that it is also a difference between two measures (eg, a specific SR and the sample mean as reference as used herein) but the residual is an Euclidean difference as per Equation 8 but not the squared difference as for Equation 6 that takes into consideration the sample variance–covariance matrix. Residuals here are, thus, simply dioptric differences of two powers for each participant concerned and we are directly comparing each SR to the mean SR.

As for Mackenzie,^11^ sample size estimation for reproducibility studies was performed using the International Organisation for Standardisation, 1994 recommendations to determine the range from −*P* to *P* within which an estimate of SD (*s*) in relation to the population standard deviation (*σ*) of a sample of independent measurements would fall with a 95% level of probability where:

(9) $$P(-A_{R}§amp;lt;\frac{s-\sigma }{\sigma }§amp;lt;A_{R})=P$$

and

(10) $$A_{R}=1.96\sqrt{\frac{{p\left[1+n(\gamma^{2}-1)\right]}^{2}+(n-1)(p-1)}{2\gamma^{4}n^{2}p(p-1)}}$$

In Equation 10, *n*=1 is the number of independent measurements (per eye per optometrist), *p* is the number of optometrists sampled (here 50) and γ (=2; see MacKenzie^11^ is the estimated ratio (2 to 1—see Mackenzie^11^ of reproducibility to repeatability for *s*. Sampling from 50 optometrists would produce an estimated *s* within 19.8% (or approximately 20%) of the population *σ* with a certainty of 95%. Doubling the number of optometrists would only reduce this uncertainty by about 6% ( $A_{R}\approx 14\%$ ) but would increase study costs significantly and, thus, it was decided to base the study on 50 optometrists as this would also facilitate comparisons with a similar study in the UK by MacKenzie of 40 independent optometrists and one near-emmetropic participant.

## Results

### Stereopair scatter plots, surfaces of constant probability density and excesses of individual SR over mean SR

**Figure 1:**
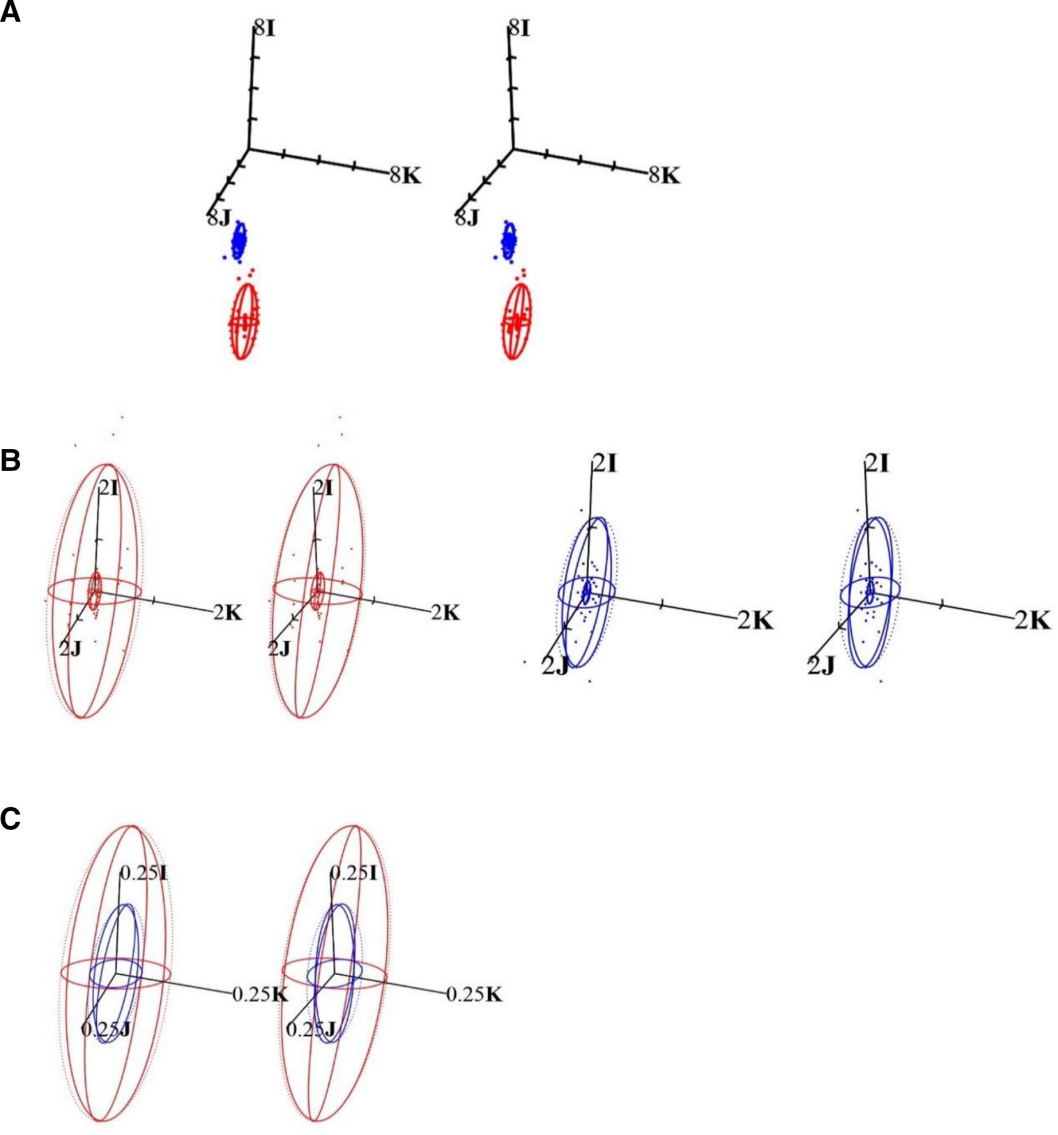
Stereo-pair scatter plots (A) for subjective refraction (SR) for the right and left eyes of a single participant as measured by 50 optometrists. Red and blue are used for OD and OS, respectively, and 95% surfaces of constant probability density are included. The sample for OD is more variable and more myopic and astigmatic (see table 1 also) but possible outliers (points outside of the surfaces of constant probability density (SCPD)) are noted for both samples. The axis lengths are 8I D with tick intervals of 2I, 2J and 2K and the origin is at O D, which is emmetropia. In clinical terms, axis length and tick intervals are 8 D and 2 D with the origin at 0 D. (B) The excesses ( $=SR-\overset{-}{SR}$ _OD_ for OD or $=SR-\overset{-}{SR}$ _OS_ for OS for each optometrist) are indicated using points with corresponding 95% distribution ellipsoids (larger) and confidence ellipsoids on the means (smaller, near the origins and difficult to see; there are two ellipsoids in each part of B)). Note the changes in scale. In (C) the excesses (the points) and the two distribution ellipsoids are not included, thus the 95% confidence ellipsoids on the means (CEM) are much easier to observe with the modified scale. The origins in (B) and (C) represent an excess of 0 D, that is, the 2×2 null matrix. (In clinical or conventional terms, this is equivalent to a null excess or 0 D.).

The means for SR for the right and left eyes of the participant in scientific (matrix **F** and vector **f**) and clinical notation are included in table 1 and the corresponding samples are plotted with 95% distribution ellipsoids (surfaces of constant probability density; SCPD) in figure 1. Variance–covariance matrices for SR for the right and left eyes of this participant are shown in table 1. The variances are along the diagonal (top-left to bottom-right) and covariances are off-diagonal (upper and lower entries). Only six numbers are distinct in these symmetric variance–covariance matrices.

**Table 1:** Non-cycloplegic subjective refractions (SR) were independently measured for each eye by 50 experienced optometrists (>5 years)

	Right eye	Left eye
**Means**: **F** (D or m^-1^)	$\left(\begin{array}{cc} -7.816 & 0.770\\ 0.770 & -12.044 \end{array}\right)$	$\left(\begin{array}{cc} -4.592 & -0.064\\ -0.064 & -6.438 \end{array}\right)$
**Means: f (D or m^-1^**)	${\left(-9.9282.1220.747\right)}^{T}$	${\left(-5.5080.9230.077\right)}^{T}$
**Means** ( $\overset{-}{SR}$ ):	$\begin{array}{ccc} F_{S} & F_{C} & A\\ -7.68 & -4.50 & 10 \end{array}$	$\begin{array}{ccc} F_{S} & F_{C} & A\\ -4.59 & -1.85 & 178 \end{array}$
Clinical notation (D, D, °)		
**Variance–covariance matrices** (D^2^)	$\left(\begin{array}{ccc} 0.381 & -0.235 & -0.024\\ -0.235 & 0.331 & 0.069\\ -0.024 & 0.069 & 0.093 \end{array}\right)$	$\left(\begin{array}{ccc} 0.162 & -0.004 & 0.012\\ -0.004 & 0.026 & -0.003\\ 0.012 & -0.003 & 0.015 \end{array}\right)$
**Volumes (D** ^**3**^ **)**		
95% distribution ellipsoids	18.952	1.976
95% confidence ellipsoids	0.064	0.007
**Mean excesses**		
Scientific notation (D)	–0.0000**I**+0.0000**J** – 0.0000**K**	0.0000**I** – 0.0000**J** – 0.0004**K**
Clinical notation (D, D, °)	–0.00–0.00×145	0.00–0.00×129
**Norms or magnitudes of mean excesses** (D)	0.000	0.000

The sample means are given using three methods, namely, using dioptric power matrices (**F**)—see Equation 1 above, transposed coordinate vectors (**f**)—see Equation 5 for the same but as column rather than row vectors, and standard or clinical notation (*F*_s_ *F*_c_ *A*). Variance-covariance matrices and the volumes for 95% surfaces of constant probability density (SCPD), that is, 95% distribution and confidence ellipsoids on means (CEM) are also included. Excesses (see later) of SR and the criterion-standard, either $\overset{-}{SR}$ _OD_ or $\overset{-}{SR}$ _OS_ as applicable, are also included.

In table 1, the stigmatic variance (*S*_II_=0.381 D^2^) for the right eye (OD) is slightly larger than the orthoantistigmatic variance (*S*_JJ_=0.331 D^2^) and much larger than the oblique antistigmatic variance (*S*_kk_=0.093 D^2^). Two of the three covariances are near zero, with *S*_JI_ = −0.235 D^2^ for the scalar and orthoantistigmatic coefficients of power. This means there is little to no linear relationship between the scalar and the oblique antistigmatic variances, but there might be some relationship between the scalar and the orthoantistigmatic variances. So, these covariances allow for understanding of variation along the three axes of stereopairs as in figure 1 and whether variation along these axes in SDPS is effectively independent (unrelated) or related. The samples and orientations of the ellipsoids may be related to such covariances as well as other factors such as ocular accommodation. For SR for the left eye (OS), there is mainly scalar or stigmatic variance (0.162 D^2^) with very little antistigmatic variance and the covariances are very close to zero with *S*_IK_=0.012 D^2^ being the furthermost from zero (meaning 0 D^2^ or no linear relationship). Note the closer alignment of the blue ellipsoid (see figure 1) with the scalar axis as compared with the red ellipsoid for OD that is more tilted away from the scalar axis.

Plotting refractive states (SR) as points on a set of three mutually orthogonal axes produces a three-dimensional scatter plot of SDPS (as in figure 1). The scalar axis, *F*_I_ **I,** is labelled 8**I**, and this axis includes all possible scalar or spherical powers although only a small part of the axis is included in figure 1. JCCs (antistigmatic powers) are represented by the *F*_J_ **J** and *F*_K_ **K** axes, which are orthogonal to the scalar axis. The orthoantistigmatic (*F*_J_ **J**) axis represents JCC with principal meridians of 90° and 180° and JCC with principal meridians of 45° and 135° are represented by the oblique-antistigmatic (*F*_K_ **K**) axis. The distributions for SR for the right (in red) and left (in blue) eyes and their distribution ellipsoids contain 95% of measurements for the participant about the sample mean (at the centre or centroid of the respective ellipsoid concerned). The volumes for the ellipsoids were 18.952 D^3^ and 1.976 D^3^ for the right and left eyes, respectively, and the larger variances (see table 1) and ellipsoid for OD indicates less reproducibility for SR in comparison with that for OS. If all SR were equal for an eye (OD or OS) here, then only a single red and single blue point would be present and each SCPD would also degenerate to a point and, thus, the spread of the points and sizes of the ellipsoids are indicative of the levels of variability and reproducibility for the samples concerned. Thus, the smaller the ellipsoids and the tighter the clusters of measurements, the greater the reproducibility. If a limited number of outliers were removed from these samples, both ellipsoids would become smaller and reproducibility for SR for that eye concerned would also improve. Volumes and variances would similarly decrease with removal of outliers.

SCPD (or 95% distribution ellipsoids) as in figure 1 are used to estimate the spread of data around the sample mean and the centroid or centre of the ellipsoid indicates the estimated mean of the sample. Essentially approximately 95% of the sample are included within the ellipsoid concerned and should the sample be representative of the population, then the same would apply to the population. One can also estimate SCPD for sample means rather than the sample data (measurements). So, the 95% distribution ellipsoids in figure 1 are, thus, estimates for regions that include 95% of the 50 measurements for the eye (OD or OS), whereas 95% confidence ellipsoids are estimates for the means of the samples (again for OD or OS). The shapes, orientations, maximum and minimum diameters and the sizes and volumes for these ellipsoids provide further information about the nature or distribution of the sample and its variation.

### Excesses (differences)

The mean SR for OD (or OS) was subtracted from the individual SR for each optometrist involved and the differences or excesses in refractive state in relation to the mean SR for the right and left eyes were indicated (figure 1B).

For the excesses (ie, of SR for OD and OS with $\overset{-}{SR}$ _OD_ and $\overset{-}{SR}$ _OS_ as applicable), there are very small 95% confidence ellipsoids (figure 1C) on the means that are located very close to the origins (an excess of zero or **O** D) of the stereopairs. Their centroids (see table 1) are the mean excesses for OD and OS and are almost zero for the 50 optometrists even though there are possible outliers (optometrists that had large differences or excesses between their SR and the mean SR ( $\overset{-}{SR}$ )). There are also 95% distribution ellipsoids in figure 1B that are much larger than the 95% confidence ellipsoids. The larger red ellipsoid for the sample for the right eye (in figure 1C) is very slightly tilted away from the scalar axis and the blue ellipsoid for the sample for the left eye probably relates to differences in astigmatism in the sample for the right eye as against that for the left eyes with possibly one or more excesses that might be considered outliers (note the red points outside the red ellipsoid in figure 1A, B and some points are further located from the scalar axis in relation to that for the blue points that are mostly closer to the scalar axis; so, greater variation in astigmatism for OD as against that for OS). The variance–covariance matrices in table 1 correspondingly indicate greater ortho- and oblique antistigmatism (and astigmatism) for the sample for the right eye (0.331 and 0.093 D^2^) in comparison with that for the left eye sample (0.026 and 0.015 D^2^).

Note the ellipsoids in figure 1 mainly vary about the scalar axis (and the thickness perpendicular to that axis or indeed in any direction relative to the scalar axis is small; mean excesses are, thus, primarily scalar or spherical with very little astigmatic or antistigmatic variation). The smaller an ellipsoid in figure 1C, the greater would be the agreement and similarity between the 50 measurements for SR and the criterion-standard, either $\overset{-}{SR}$ _OD_ or $\overset{-}{SR}$ _OS_ whichever might be applicable. Although the ellipsoids in figure 1C seem quite large, the scale is only 0.25 D in clinical terms. Thus, agreement and reproducibility for SR across the 50 optometrists are good to excellent.

In terms of scalar powers, a negative excess (and point below the origin in figure 1B) would indicate that the optometrist concerned found more negative power (or potentially over-minused) the SR for the participant.

### Normality

**Figure 2:**
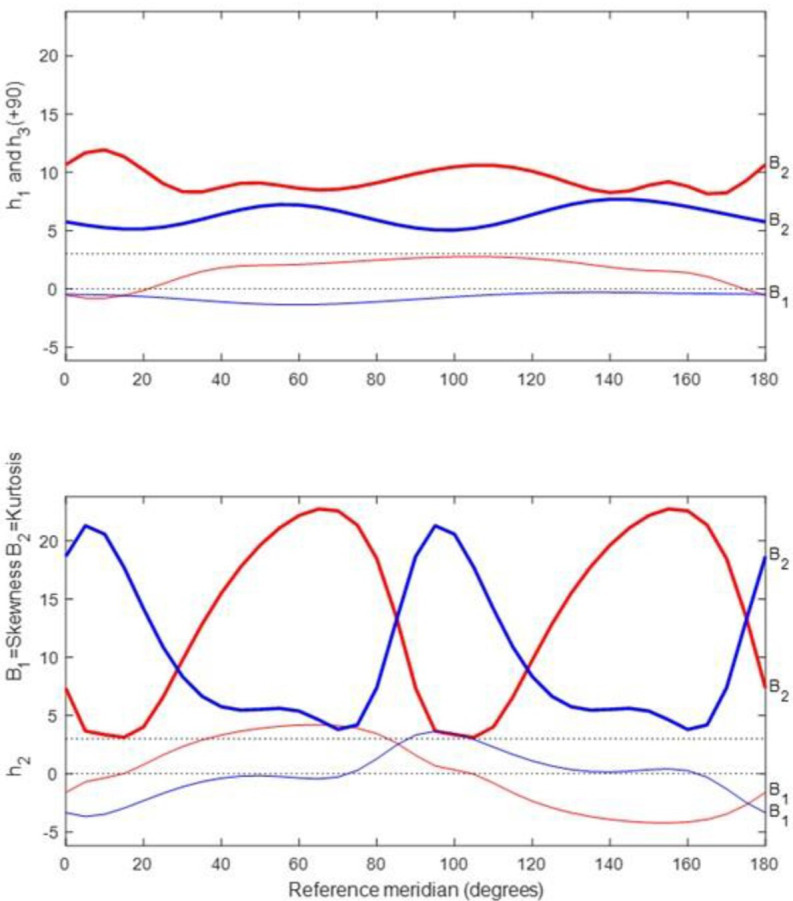
Meridional profiles for kurtosis (thicker) and skewness (thinner) for subjective refraction (SR) from 50 optometrists for the participant. Profiles for curvital (h_1_ and h_3_) and torsional powers (h_2_) are at the top and bottom, respectively, and red and blue are used for OD and OS as for previous plots.

To investigate the normality of the data, profiles for univariate skewness (*β*_1_) and kurtosis (*β*_2_) were plotted (see figure 2). The thicker curves indicate kurtosis (labelled *β*_2_) while the thinner curves indicate skewness (*β_1_*). There are also two dotted lines on the graph representing values of zero (no skewing) and three (3) for kurtosis of dioptric powers, which indicates mesokurtosis. In figure 2, for OD and OS for SR, there is profound leptokurtosis (between 5 and 20—see the thicker profiles—depending on meridian in the top plot and 1 to >20 in the bottom plot that may relate to possible outliers or other factors) and positive and negative skewing (as shown by the thin red and blue profiles in figure 2).

One can also evaluate normality with a multivariate approach^32^ and for trivariate normality with SR, skewness (*b*_1_, 3) should be zero, and for a mesokurtic sample, kurtosis (*b*_2_, 3) should be 15 (p=3 and p (p+2)=3 (3+2)=15) provides the expected value. For trivariate samples, such as dioptric powers, values below and above 15 indicate platykurtosis and leptokurtosis, respectively. So, Mardia’s multivariate statistics^32^ also indicate that there was positive skewness (*b*_1_, 3 is 27.650 and 15.086) and leptokurtosis (*b*_2_, 3 is 46.597 and 35.850) for the right and left eye, respectively, of the participant concerned.

For figure 2, coordinate vector **h** from Harris^13^ is used and the following equation is relevant:

(11) $$h=\left(\begin{array}{c} h_{1}\\ h_{2}\\ h_{3} \end{array}\right)=\left(\begin{array}{c} f_{11}\\ {\sqrt{2}f}_{21}\\ f_{22} \end{array}\right)$$

Coordinate vector **h** can be obtained from clinical notation with:

(12) $$h_{1}=F_{s}+F_{c}sin^{2}A$$

(13) $$h_{2}=F_{s}-F_{c}sinAcosA$$

(14) $$h_{3}=F_{s}+F_{c}cos^{2}A.$$

Given that both the univariate and multivariate analyses indicated departure from normality and outliers were also possible, caution should be exercised with interpretation of variances and covariances (table 1) and the SCPD as in figure 1. One might remove atypical measurements (outliers) from some of the optometrists and then reconsider the various plots and statistics to see whether the samples would shift towards greater normality. MD were used to identify possible outliers for SR for the right and left eyes of the participant (see Eq. 6 and the online supplemental figure to this paper). Although most MD were <2, three measurements (from optometrists 2 and 5 for the right eye and optometrist 10 for the left eye with relatively large MD of ≈5 to 6) were possible outliers (with almost 90% probability that they were outliers).

### Polar profiles of variance for dioptric power

**Figure 3:**
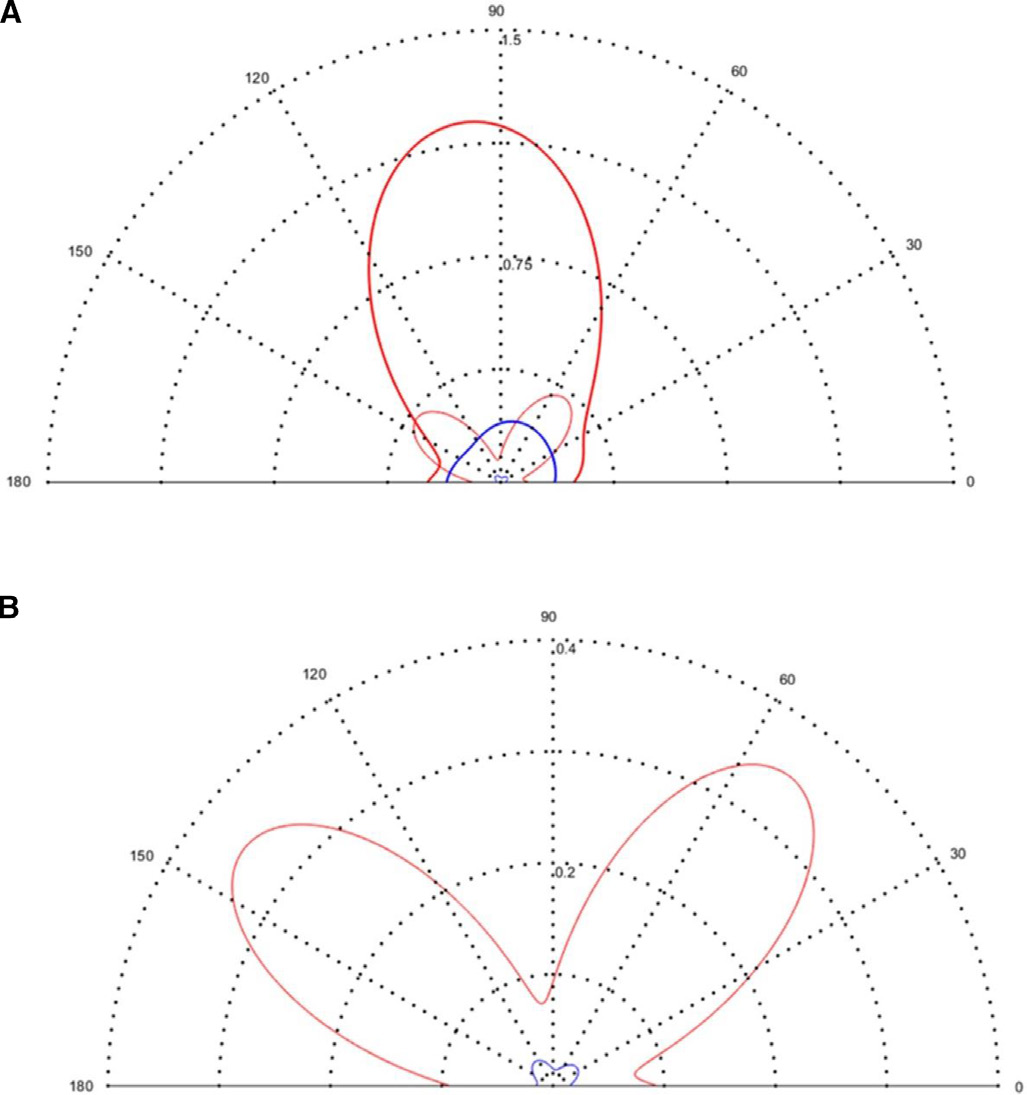
Polar profiles for curvital and torsional variance for subjective refraction (SR) for the right (in red) and left (in blue) eyes of the participant as measured by 50 optometrists. (A) Thicker and thinner profiles represent curvital and torsional variances respectively. The radial scale ranges from 0 to 1.5 D^2^ with intervals of 0.375 D^2^. (B) The radial scale is changed to 0 to 0.4 D^2^ with intervals of 0.1 D^2^ for easier observation of the torsional profiles.

Polar profiles^27^ as in figure 3 depict three variances (for the curvital and torsional coefficients of dioptric power) with the meridional scale representing reference meridian (*θ*) in degrees from 0 to 180°. The polar origin is 0 D^2^ (ie, no variance) and the radial scale represents the magnitude of the variance. The closer a polar profile is to the origin, the smaller is the variation for that coefficient of power. The black dotted semicircles from the polar origin represent change in magnitude of variation as per values indicated along the vertical meridian (90°). The thicker solid profiles in figure 3A represent the curvital variances of *f*_11_ (=*h*_1_) and *f*_22_ (=*h*_3_), which are both indicated on a single profile but with a 90° phase difference. The thinner solid profiles are for the torsional variance of *f*_12_ = *f*_21_ (= $\sqrt{2}$ *h*_2_). A semicircular curvital profile of constant radius for *f*_11_ and *f*_22_ and torsional variance of 0 D^2^ for all meridians indicate purely stigmatic or spherical variation and in the case of non-uniform variation, the torsional profiles appear to resemble ‘rabbit ears’ with the symmetry of the ears always being 90° apart.^27^ The symmetry also allows one to determine the minima and maxima for antistigmatic variance.

In figure 3A, there is greater curvital variance for the right eye (thick solid red profile) with a maximum variance (≈1.25 D^2^) just beyond the vertical meridian near 100°. The left eye has maximum curvital variance (thicker solid blue profile) of ≈0.25 D^2^ near 70°. For both OD and OS, torsional variances are mostly less than the curvital variances irrespective of meridian (figure 3A). Variance–covariance matrices (table 1) only indicate the variation along the stigmatic, orthoantistigmatic and oblique-antistigmatic axes of a stereopair scatter plot, whereas polar plots for variance^27^ indicate variation for all meridians and are, thus, a more complete representation of variation for the samples concerned.

### Reproducibility via starbursts and Euclidean distances

Reproducibility for SR for the right and left eyes of this participant can be assessed with starbursts and residuals (excesses or differences) of SR from each optometrist as compared with the mean SR ( $\overset{-}{SR}$ ) for the group of optometrists (N=50). The $\overset{-}{SR}$ for the right or left eye is regarded as the standard for comparison and the SR measured by each optometrist is subtracted from the right or left $\overset{-}{SR}$ using matrix methods.

**Figure 4:**
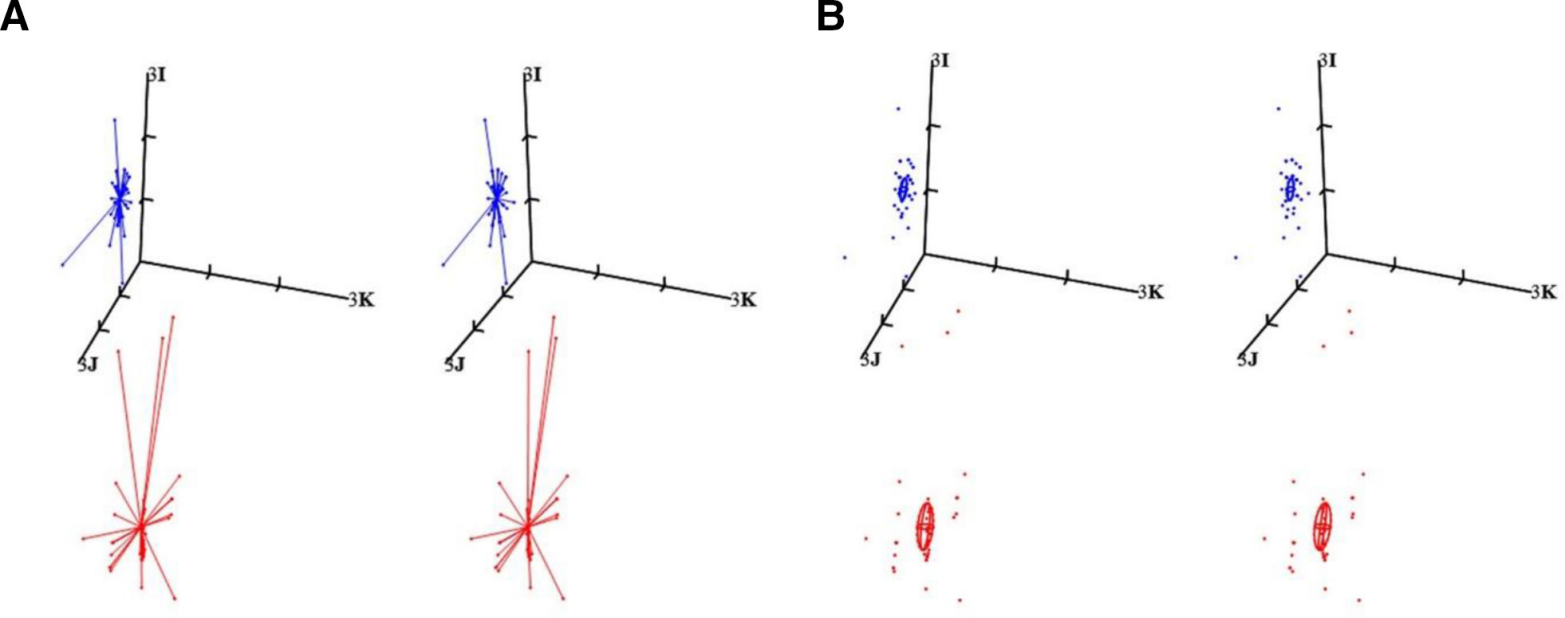
Stereopairs are used for (A) starbursts and (B) SR from 50 optometrists and mean subjective refraction (SR) ( $\overset{-}{SR}$ ) for the eyes (OD and OS) concerned. Confidence ellipsoids (95%) for the sample means are also included for both eyes. Red and blue are used for the right and left eyes respectively. The origin is the matrix, −7I D (or −7 D in clinical terms) in both (A) and (B). Axis (3I D) and tick intervals are the same in both parts. In clinical terms (*F*_s_ *F*_c_ *A*), $\overset{-}{SR}$ _OD_ = −7.68–4.50×10 and $\overset{-}{SR}$ _OS_ = −4.59–1.85×178 (as indicated in table 1). After transformation to matrix notation, these are the values at the centres of the red and blue starbursts for the right and left eyes, respectively, of the participant. For both the right and left eyes, the stereo-pair in (B) includes the mean subjective refraction ( $\overset{-}{SR}$ _OD_ or $\overset{-}{SR}$ _OS_) and individual SR for each eye as applicable. The sample mean (at the centroid of the ellipsoid concerned) is our best estimate of the true or population mean for SR for the eye concerned.

Stereopair starbursts (figure 4) connect the individual subjective refractions (N=50 per eye) to the applicable sample mean (for either the right or left eye) at the centre of the starburst. The subjective refractions by the 50 optometrists are each indicated by dots at the ends of the lines (comets) that all meet at the centre, which is $\overset{-}{SR}$ for the 50 subjective refractions for either OD or OS. The mean subjective refractions in clinical notation are –7.68–4.45*×*10 for the right eye (red starburst) and –4.59–1.85×178 for the left eye (blue starburst). This is the criterion or gold standard that is used to compare with each SR from the 50 optometrists. So, the shorter a line or comet in the starburst, the closer the SR by that optometrist was to the mean subjective refraction, $\overset{-}{SR}$ . The blue starburst (for the left eye) is smaller with shorter comets with a few exceptions rather than the larger red starburst and longer comets for the right eye, where optometrists deviated further from the criterion-standard $\overset{-}{SR}$ . The sample means for the subjective refractions for OD or OS are, of course, related to their respective population means (that are unknown).

If all 50 optometrists had the same result (SR) as the sample mean, then the starburst for that eye (right or left) would collapse or degenerate to a single point at the centre of the relevant starburst. The red starburst (for OD) is further away from the stigmatic axis (with label 3**I**) since the cylinder is greater in the right eye (*F*_c_ = – 4.45 D) than in the left eye (*F*_c_ = –1.85 D).

The Euclidean differences (see Equation 8 and reference 13, although here coordinate vector **f** and ||**f**_q_ – **f**_p_|| is used rather than coordinate vector **h** and

||**h**_q_ – **h**_p_||) for a starburst which compares the individual subjective refraction (**f**_q_) for each optometrist to the mean subjective refraction (**f**_p_ = $\overset{-}{SR}$ _OD_ or $\overset{-}{SR}$ _OS_ for OD or OS, respectively) for the sample of 50 optometrists were calculated and they represent the dioptric lengths of the comets making up a starburst. Most of the 50 optometrists concerned were within 0.60±0.65 D (mean Euclidean differences $\left(||\overset{-}{ED}||\right)$ ±SD for Euclidean differences ( $s_{ED}$ )) for the right eye and 0.34±0.29 D for the left eye of the mean subjective refractions for OD and OS for the participant involved. The ranges for the Euclidean differences for OD were 0.08 to 3.37 D and for OS, 0.12 to 1.36 D. Reproducibility was in general better for SR for OS as compared with OD and issues such as possible outliers, departures from sample normality, learning effects and the possible greater difficulty in measuring SR for the eye with more severe ametropia may be relevant.

## Discussion

Figure 1 shows the stereopair scatter plots for subjective refractions (SR) from the 50 optometrists for the right (red) and left (blue) eyes of the participant together with their 95% SCPD. The measurements for right eye (red in figure 1) were more variable and more myopic and astigmatic (see table 1 also). Each point in the figure represents the SR from a single optometrist (of the 50 involved) for the participant’s right or left eyes. The boundary of the distribution ellipsoid describes the region of the 3-space (SDPS) within which an estimated 95% of the sample (ie, SR) and possibly the population also from which the sample was drawn based on assumptions such as normality of data and random sampling. In this instance due to outliers (measurements of SR that were atypical for the sample) and departure from normality as well as the purposive nature of sampling of the optometrists rather than a random selection, we need to be cautious when interpreting the meaning of these SCPD for the right and left eyes. Nonetheless, the points themselves and their distributions in SDPS are important in terms of measures of central tendency (ie, means) and measures of dispersion (such as variances) and the points (measurements) alone are not subjected to the abovementioned assumptions (normality and random sampling) for the ellipsoids. Quantitative measures for variances and covariances will be influenced by factors such as outliers and departure from normality. The centres (or centroids) for these distribution ellipsoids represent the sample means (–7.68–4.50×10 and –4.59–1.85×178 for the right and left eyes, respectively; see table 1). Means such as these are more robust to outliers but where necessary medians can be used instead.

All points below the origin of the three axes in figure 1A indicate the myopic astigmatic states of the eyes concerned. The cluster of points in blue closest to the origin represents SR for the left eye. The points lying outside the ellipsoidal boundary or surface are possible outliers, and some are noted for both eyes (red for right and blue for left eye). MD and Euclidean lengths are helpful in identifying the optometrists where SR was atypical as against the remainder of the group. These would be the ones with the largest Euclidean lengths (or differences) for OD and OS. However, most of the optometrists had similar results (if we exclude a few that were atypical), but there was possibly greater difficulty with measurement of SR in the eye with the greater ametropia (ie, the right eye of the participant).

The excesses in figure 1B and mean excesses in table 1 also confirm that despite a few larger excesses (possible outliers), mostly SR is highly reproducible across the 50 optometrists concerned and with learning and experience by the participant reproducibility seems to improve also. Variation in the residuals was mainly scalar (spherical) rather than antistigmatic as indicated also in table 1. The starbursts in figure 4 and the mean Euclidean differences again indicate the high reproducibility of SR and despite the presence of moderate to severe ametropia in the eyes of the participant involved. As before, greater difficulty was experienced in measuring SR for the eye with greater ametropia and the mean Euclidean difference was almost double that for OD (=0.60 D) as against that for OS (=0.34 D). SD for the Euclidean differences was also greater for OD, that is, for the eye with greater ametropia.

There is minimal variability in the scalar coefficients of SR (see table 1 where *S*_II_ ≈0.38 D^2^ and ≈0.16 D^2^ for OD and OS, respectively), with again greater variance for SR for the eye with greater ametropia. Note also the relatively large orthoantistigmatic variance (*S*_JJ_ ≈0.33 D^2^) for OD as compared with the other antistigmatic variances). The differences in SR findings could be due to changes in the participant’s subjective responses (perhaps because of eyelid squinting or misunderstanding instructions and the examiners using different refractive procedures or different endpoint criteria). Fatigue and/or learning may also factors depending on circumstances during specific SR and, of course, relating also to the techniques of individual optometrists and their ease of measuring the SR. Since the participant was prepresbyopic and, thus, still able to accommodate, some optometrists might have failed to completely relax the participant’s accommodation and, thus, this might have been a confounding or extraneous factor. Since all the visits were completed within a period of 6 months, it is unlikely that the participant’s subjective refractive states would have changed very much between the various examinations or that other factors such as diurnal variation (SR was measured at different times of day) or environmental such as involving ambient temperature or humidity would have been that influential although one cannot obviously rule out some effects of this sort. While some optometrists might have used ‘maximum plus to best visual acuity’ as their endpoint, others might have chosen other options (undercorrecting or overcorrecting slightly) and this is, thus, an unknown factor in terms of the final SR that each optometrist determined and possible influences in this study.

Of importance, here is that besides possible outliers, the samples for SR were also not found to be normally distributed and the samples mainly show leptokurtic distributions for SR irrespective of eye (OD or OS) concerned, but there is also marked or severe positive or negative skewing depending on meridian concerned (figure 2). Some of this departure from normality relates to the presence of possible outliers or variability due to the various methods applied during measurement of SR and perhaps even differences in application of interocular accommodative equalisation or balancing techniques (such as Humphrey immediate contrast test or others), for example, or differences in endpoints and possibly also different experience levels for the optometrists concerned. Increasing the sample size and randomisation in selection of optometrists possibly might reduce such departure from normality and be helpful, but it could also be that SR is inherently not normally distributed due to processes (such as emmetropisation or departures therefrom and genetics) that apply to ametropia, and it is understood that some samples such as that for university students are typically leptokurtic and skewed with a greater prevalence of myopia.^35 36^

Although an analysis such as herein must first consider and understand the samples themselves, the research question here is directed towards exploring and understanding reproducibility for SR for the right and left eyes of the participant and the last section of the results (see above) analyses this aspect, mainly through the use of starbursts (figure 4), and also Euclidean differences^13^ and excesses (see figure 1B and C and table 1) for SR from each optometrist as compared with the means for SR for OD and OS from the 50 optometrists. The mean SR for either the right ( $\overset{-}{SR}$ _OD_) or left ( $\overset{-}{SR}$ _OS_) eye was regarded as the gold-standard or criterion-standard, and the SR measured by each optometrist was subtracted from the mean SR for the right or left eye as applicable using matrices. Euclidean differences compare the individual SR for each optometrist to the mean SR for the 50 optometrists. If an optometrist had the same result as the mean SR for the eye concerned, then the Euclidean difference would be zero and the larger the corresponding Euclidean difference, the farther away that optometrist was from the mean SR for either the right and left eyes. So, figures 1 and 4 are relatively simple methods to visually understand SR for the different eyes and different optometrists involved while table 1 supplies quantitative results to indicate just how different are the measurements, that is, concerning the level of agreement or similarity and reproducibility of SR.

The results for this study indicated that most optometrists were within 0.60±0.65 D of the mean SR for the right eye and 0.34±0.29 D for the left eye (here, mean Euclidean distance $||\overset{-}{ED}$ || and SD ( $s_{ED}$ )). These are measures in SDPS and they are always positive; they are also not quite the same as other measures for reproducibility that might be expressed in terms of clinical notation or power vectors although they can be thought of in *sphere-equivalent* terms. The smaller the value and closer to zero is $||\overset{-}{ED}$ ||, the greater is the reproducibility for SR and in terms of the SD here, the smaller the value, the lesser the variation of the Euclidean differences. Here, the values ( $s_{ED}=$ 0.65 D and 0.29 D are relatively large (in relation to their means) but removal of possible outliers would decrease $s_{ED}$ . So, this study suggests that SR performed by 50 optometrists on a single participant may differ (sphere-equivalent mean Euclidean differences) by ≈0.34 to 0.60 D. The ranges for the Euclidean differences for the right eye were larger than for the left eye, meaning that the reproducibility was better for SR for the left eye with the lesser ametropia as compared with the right eye. This could be due to issues such difficulties in performing SR or less experience perhaps with some of the optometrists that resulted in the possible outliers (eg, optometrists 2, 5 and 6 for OD and 5, 9 and 10 for OS). These optometrists (see online supplemental figure) had larger Mahalonobis distances and in the starbursts longer lines or comets correspond to these observations. (These potential outliers can also be found in figure 1A outside the distribution ellipsoids for OD and OS.)

Excesses (table 1 and figure 1) are used to compare each SR for OD or OS of the participant to the means ( $\overset{-}{SR}$ _OD_ or $\overset{-}{SR}$ _OS_) for the group of 50 optometrists. Of course, we do not know the true SR for OD or OS for the participant and, thus, the averages for SR for the group are used as our best estimates of the true SR for OD and OS. These means are, thus, the references to which individual subjective refractions are compared and the smaller the residual (excess or difference), the closer is the optometrist measured SR to the mean SR. Although there were some optometrists with larger excesses, where the SR was quite different to the mean SR for the eye (OD or OS) concerned, the mean excesses in table 1 were almost zero and thus, on average, there was not too much of a difference between results for individual optometrists for SR as compared with the mean SR for either OD or OS and consequently the 95% confidence ellipsoids (most obvious in figure 1C) are very small in size and positioned very close to **O** D (or 0 D in conventional terms).

MacKenzie,^11^ in the UK, investigated the reproducibility of spherocylinder prescriptions from a healthy young man as provided by 40 experienced optometrists. His study also used univariate and multivariate methods for dioptric power to evaluate the reproducibility. He concluded that optometrists may differ in their stigmatic component (*F*_I_ or *M*) by ≈0.78 D and approximately 0.50 D cylinder (*F*_c_) in 95% of repeated measures. The current study mainly used Euclidean differences,^13^ which can be either sphere equivalent or cylinder equivalent^13 37–39^ to obtain the 95% of repeated 39 measures. Sphere equivalency was used herein as this is possibly easier to understand in terms of clinical applications and we found similar results to MacKenzie. In another study by Shah *et al*,^40^ six eyes from three groups of *standardised* patients (basically patients trained to be expert observers) with healthy eyes were investigated by three or some by four optometrists. Both the first and second groups of the standardised patients had no cylindrical correction in their right eyes and relatively small amounts of astigmatism (*F*_c_: −0.25 D) in their left eyes or no astigmatism at all. The spherical ametropia ranged from −3.75 to −4.00 D with a mean of −3.94 D. The third group had no distance prescription. Based on the reproducibility limit data obtained, they concluded that any two optometrists could differ in their spherical equivalent refraction by ≤0.75 D and between 0.25 D and 0.61 D for their cylindrical components (*F*_c_) in 95% of repeated measures.

The findings of the present study are also mostly in an agreement with those by Bullimore *et al* 8 who reported reproducibility limits for spherical equivalent refraction to be 1.10 D and 1 D for cylinder (*F*_c_). They used power vectors *M*, *J*_0_ and *J*_45_ rather than power matrices to evaluate their results, but their study design was based on examination of 86 participants by two examiners, so comparisons of their results and ours (one participant only but 50 examiners) should be made with caution as the research designs were obviously quite different. Based on the limits of agreement, Zadnik *et al* 7 estimated the 95% limits of agreement for SR to be ±0.63 D. However, their study findings are not very comparable with the results of our study since their findings were based on an analysis of power measured along the vertical meridian of each eye. Rosenfield and Chiu^10^ investigated the repeatability of subjective and objective refractions by one examiner on 12 participants on five separate occasions and showed that 95% limits of agreement for sphere (*F*_s_) and cylinder (*F*_c_) powers were ±0.29 D and ±0.16 D, respectively. However, their study assessed repeatability (repeated measures by the same examiner on different occasions; in their case, two examiners analysed separately) rather than reproducibility (different examiners as compared with one another) as investigated in the current study.

While there are several studies^2 5–11^ that provide insight into the reproducibility of SR, the findings of those studies are based on SR measurements collected from two, three or even five examiners, and in some instances, students are used as participants. Another limitation was that examiners were not always masked to the results of previous SR or spectacle prescriptions. Although the present study investigated the reproducibility of the SR of a symptomatic participant using SR from 50 experienced optometrists, this participant cannot be considered as representative of the diverse population of South Africa or of the range of ametropias possible. Other possible limitations relate to the methods used for measurement of SR that might have varied across the different optometrists and their years of experience after graduation varied from 5 to 20 years. Environments or practices where the SR were measured would also differ in terms of lighting and different charts and test distances might also apply. Mostly phoropters would have been used, but sometimes, trial frames and lenses might have been used for the full SR or to check the phoropter-based SR. Retinoscopy and/or autorefraction might also have been incorporated before doing SR. Endpoints for SR might have differed across the optometrists. All SR were measured without cycloplegia, and, thus, changes in ocular accommodation may have had unknown influences on SR as determined and analysed here. Additionally, it is likely that there may have been learning effects as the participant experienced multiple eye examinations, and this could have affected the reproducibility for SR.

Advantages for the study here include that a comprehensive investigation and analysis were performed using appropriate methods for analysis of refractive state and it is probably the first study of this type in the South African context. Reproducibility of SR is not really that well understood and this study advances our knowledge and understanding of this rather complicated but intriguing topic.

### Recommendations

Future studies could include more participants and perhaps fewer optometrists and possibly be performed both with and without cycloplegia. A wider range of ametropias could be useful in developing greater understanding of the area of interest involved. Younger and older participants than the one involved here would also be very useful for further study. This study was based in only a single geographic region and similar studies could be done elsewhere. A standard protocol for SR could be developed and used by the different examiners involved, and greater control of clinical environments could be used in future studies of this type should the intention be to limit specific factors that might affect reproducibility. Learning effects also should be taken into consideration where multiple eye examinations are contemplated.

## Conclusions

Absolute 95% reproducibility limits suggested that SR performed by multiple optometrists (N=50) on this participant differed for scalar powers by ≤1.71 D for the moderately to highly myopic astigmatic right eye and by ≤0.75 D for the moderately myopic astigmatic left eye. Removal of a few possible outliers from OD and OS, respectively, reduces the 95% reproducibility limits for the scalar powers to ≤0.97 D (and the antistigmatic coefficients from ≤0.69 D to ≤0.49 D), thus improving interexaminer reproducibility. In other words, after removing a limited number of possible outliers, subjective refractions performed by multiple optometrists on this participant with ametropia differed in their scalar coefficient (*F*_I_=*M*) by up to ≈1 D and their antistigmatic coefficients (*F*_J_ = J_0_ and *F*_K_=J_K_) by up to ≈0.5 D. This is due to many factors including characteristics of the examiner, the participant and the method for SR and the clinical environments themselves. Less variation and greater reproducibility for the antistigmatic components (*F*_J_ = *J*_0_ and *F*_K_ = *J*_45_) were found and the same applies also to *F*_c_ that was less variable than *F*_s_. However, for eyes with smaller magnitude cylinders, it is likely that greater variation in SR across multiple optometrists would occur depending also on skill levels of examiners as well as participants and their ability to respond to the procedures during measurement of SR. Even in healthy eyes, several factors may lead to variation in SR and this paper describes such factors of importance in obtaining satisfactory measures for SR. For example, learning and exposure to multiple examinations such as for the participant in this study may have reduced reproducibility as the participant became more experienced in responding to SR over time. Reproducibility in typical practice or clinical environments might, thus, not be as good as reported here, given that patients normally would not have anywhere as many SR performed as for this participant. However, the paper emphasises the need to investigate some of these issues in further detail. Many factors such as gender, race, level of education, previous experience of both participants and examiners and others such as type and severity of ametropia may influence reproducibility of SR and, thus, could be productive areas for future investigation. This study indicated that reproducibility for SR for the participant concerned (with moderate to severe ametropia, specifically myopic astigmatism) was good to excellent even in the absence of cycloplegia or a standardised protocol for SR, varying levels of experience of the optometrists concerned and no attempt at standardisation of the environments within which the measurements were performed.

## Data Availability

Data are available upon reasonable request.
