# Supplementary material for: Interexaminer reproducibility for subjective refractions for an ametropic participant
Source: BMJ Open Ophthalmol. 2022 May 11;7(1):e000954. doi: 10.1136/bmjophth-2021-000954 (PMC9096535; doi:10.1136/bmjophth-2021-000954)
Supplement: Supplementary data [file bmjophth-2021-000954supp001.pdf]

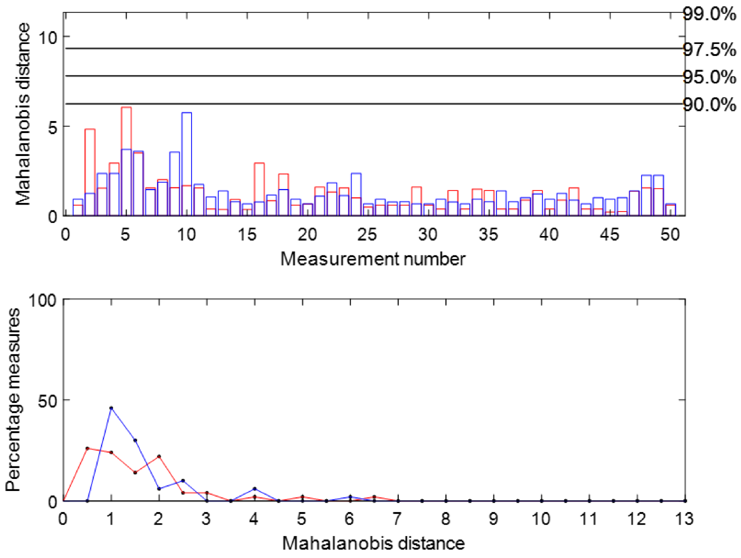

Mahalanobis distances for SR the right and left eyes of a single participant indicated using red and blue bars (top). Most Mahalanobis distances were < 2 and only two possible outliers are indicated where bars almost reach the 90% level of confidence.
